# Seasonality and nutrition-sensitive farming in rural Northern Ghana

**DOI:** 10.1007/s12571-022-01325-5

**Published:** 2022-11-23

**Authors:** Ilse de Jager, Gerrie W. J. van de Ven, Ken E. Giller, Inge D. Brouwer

**Affiliations:** 1 Division of Human Nutrition and Health, Wageningen University, P.O. Box 17, 6700 AA Wageningen, The Netherlands; 2 Plant Production Systems Group, Wageningen University, P.O. Box 430, 6700 AK Wageningen, The Netherlands

**Keywords:** Nutrient adequate diet, Linear programming, Food availability, Food affordability, Household

## Abstract

In rural sub-Saharan Africa, where malnutrition in all its forms is rife, the greatest gap between the availability of foods and the foods needed for a nutritious diet are faced during the ‘hunger season’. We investigated what rural households in Northern Ghana would need to grow to ensure year-round availability of a nutrient adequate diet or the income required to fulfil their dietary needs. We applied linear programming to model different scenarios and interventions. Our results provide three major insights. First, considering seasonality is crucial in nutrition-sensitive farming. Ensuring a nutritious diet year-round requires enhanced availability of vegetables and fruits throughout the year. Second, although staple crops do not provide the full range of essential nutrients, increasing their yields allows for a reduction of field size, freeing up space for the production of other foods belonging to a nutritious diet, such as vegetables. Third, small farms are unable to produce sufficient food to cover their needs. They depend on income both from agriculture and other sources, and the availability of types of foods on markets to meet their dietary needs. Our study shows the value of modelling the range of dietary effects from agricultural interventions in a specific context, using a local feasible nutritious diet as a starting point and taking seasonality into account.

**Supplementary Information:**

The online version contains supplementary material available at 10.1007/s12571-022-01325-5.

## Introduction

Malnutrition in all its forms is rife in sub-Saharan Africa: the only subregion with a rising number of stunted children, where micronutrient deficiencies persist and where the prevalence of overweight and obesity increases (FAO et al., 2020). In many rural and low-income settings, a large proportion of food is acquired in the ‘natural food environment’ through foraging and agriculture (Downs et al., 2020; Turner et al., 2018). Thus, although most smallholder households are net food buyers, they also depend on their own agricultural production for their dietary needs. In rural Northern Ghana, where malnutrition is widespread (FAO et al., 2020), smallholders are considered the largest component of the rural sector.

Current strategies to address hunger and malnutrition, include interventions which aim to improve production of staple crops and increase income of farming households (Brouwer et al., 2020). Agricultural interventions aim to improve production of food and cash crops or livestock by addressing a wide range of constraints, including management issues, access to improved seeds and fertilisers and extension services. Nutrition-sensitive interventions further address the underlying causes of malnutrition and aim to ensure that acceptable, diverse, nutritious and safe foods, adequate to meet the dietary needs of all people, are available and affordable at all times. For example, ensuring that farmers have the resources to produce the right foods for a healthy diet. Interventions can influence diets of smallholder households through two main pathways in their food environments: increased availability and increased affordability of food (Downs et al., 2020; Turner et al., 2018). The availability pathway aims at increased production for own consumption; the affordability pathway aims at increased production for sale at the (local) market and the extra income is used for purchasing food (Du et al., 2015).

To better understand the boundaries of what is possible in terms of on-farm production for nutritious diets, we must address three issues. First, the availability of foods across seasons is essential to achieve year-round nutritious diets. Although seasonality as a proximal risk factor for undernutrition is poorly understood, existing studies all suggest that seasonal stress is an important distal driver of undernutrition (Madan et al., 2018), resulting in depressed growth among children during pre-harvest periods and with long-term slowed growth with increasing age (Ategbo, 1993). The resilience of households is key in the effects of seasonal fluctuations in consuming a nutritious diet (Waswa et al., 2021). Most households that are vulnerable to a prolonged dry season, are also vulnerable to malnutrition. Almost 60% of sub-Saharan Africa, the region with the highest prevalence of stunted children and micronutrient deficiencies (FAO et al., 2020), has only a single cropping season and a long dry season (Ker, 1995). Therefore, the situation for these households will worsen every dry season, and temporary undernutrition will easily translate into chronic undernutrition. Especially towards the end of the dry season (often referred to as the ‘hunger period’), availability of perishable but nutrient-dense foods such as fruits, vegetables and animal source foods is limited (Devereux, 2009; HLPE, 2017). Further, prices of foods and consequently the costs of a nutritious diet increase (Masters et al., 2018) and dietary diversity decreases (Abizari et al., 2017) resulting in child growth deficits (Fentahun et al., 2018). Second, globally diets are becoming more and more homogeneous and many agricultural interventions focus on improving yields of a few cereal crops (Khoury et al., 2014) through use of improved varieties, fertilizers and irrigation, and reducing post-harvest losses. Investigating diverse crop combinations may provide insight in the potential availability of foods for nutritious diets. Third, evaluations of impact of agricultural practices on diets are generally limited to interventions such as home gardens or nutrition-sensitive agricultural interventions that include specific nutrition goals (Ruel et al., 2018). There is a urgent call for taking a healthy diet perspective for food systems transformations (Brouwer et al., 2021). Taking a healthy diet perspective as a starting point to investigate how and which agricultural interventions without specific nutrition goals may contribute to the availability of the foods needed for nutritious diets, is rarely done.

We modelled the range of dietary effects from agricultural interventions in a specific context, using a local feasible nutritious diet as a starting point and taking seasonality into account. By means of exploring this modelling method, we investigated: which crops should be grown, the minimum farm size required and the potential contribution of mainstream agricultural interventions, focusing on improving production, to provide either a nutritious diet throughout the year or the additional income to purchase foods to meet dietary needs in Northern Ghana. Our study may facilitate the prioritization of agricultural interventions, both in research, through development projects and in enabling policy, that have the greatest potential to improve availability and affordability of foods for nutritious diets.

## Methods

### Study area

We selected Karaga sub-district in the Northern Region of Ghana for this study based on the high incidence of food insecurity and malnutrition. In 2014 about 32% of children below 5 years old were stunted and 9% were wasted (de Jager et al., 2017). Karaga district has one rainy season from May till October–November. The average annual temperature is 28 °C and annual rainfall is 900 to 1040 mm. The main crops cultivated are maize, rice, cowpea and yam. The population density is relatively sparse (50–100 inhabitants per km^2^) (Franke et al., 2011).

### Dietary intake study

A dietary intake study was carried out in Karaga sub-district among 337 children of 6 to 23 months of age. Details of this study are described by de Jager et al. (2018). In summary, based on a full census and listing of households with children of 6 to 23 months old, households were divided in clusters reflecting 4 child-age groups and 100 households were randomly selected from each age group list. From each selected household, one child was selected for a 24-h dietary recall. Data was collected in July 2014, during the hunger season, by trained enumerators with a first degree in nutrition and who spoke the local language during a three-week period. Dietary data available from the Ghana Living Standards Survey (2016/17) was not used for this study because of the disadvantages from using household level data for intake data (Sununtnasuk & Fiedler, 2017). Dietary intake of the children was assessed through the mother or caretaker using a quantitative multi-pass 24-h recall (24hR) (Gibson & Ferguson, 2008). All days of the week were captured and randomly assigned to subjects to account for day-to-day variation in dietary intake. Additionally, in a structured questionnaire-based interview with the head of household information was collected for all individual household members on sex, age and physiological state (menstruation, pregnancy, lactating), information on education, occupation, sources of income, religion, total cultivated land, distance to closest market, recall on the crops produced and their estimated yields during the last year. Prices of the foods consumed were collected in a market survey.

### Characteristics of the study population

In the dietary intake study of 337 children, 40% were stunted and more than 40% had an individual dietary diversity score below 4 reflecting a nutrient inadequate diet (WHO et al., 2007). In most households, farming was the main occupation and the main source of income of both the household head and of the mother of the child. Most households had a male household head and were Muslim. Travel distance to the closest market was on average 1 h. Households cultivated on average 2.1 ha with four crops of which three were used for home consumption. Most households produced grains (97%); legumes, nuts and seeds (84%); and only 8% of households produced vegetables. Further details of the study population are described elsewhere (de Jager et al., 2018).

### Optimal nutrient adequate diet for an average household

A linear programming tool e-Optifood^©^ was used to develop optimal diets, defined as a diet that meets the nutrient requirements of a specific population and considers their habitual diet patterns and costs. The children enrolled in the dietary intake study were divided into four groups according to age and breastfeeding state: 6–8 months breastfed, 9–11 months breastfed, 12–23 months breastfed and 12–23 months non-breastfed. For our analysis, we included all non-condiment foods consumed by ≥ 5% of the non-breastfed children of 12–23 months. Optifood^©^ was used to calculate a diet that best fits the nutrient requirements of non-breastfed children of 12–23 months considering their habitual diet patterns and costs (Ferguson et al., 2006). Thirteen key nutrients were considered: total fat, total protein, iron, zinc, calcium, vitamin A, vitamin C, thiamine, riboflavin, niacin, vitamin B6, folate, and vitamin B12. Details on development of these optimised diets are described by de Jager et al. (2018) and details specific for this study, including the translation of these optimised diets to household level, in Supplementary material 1). The optimal diet per season for an average household included 206 kg of whole grains, 21.2 kg of starchy plant foods, 92.4 kg of beans, 69 kg of nuts and seeds, 14.4 kg of soybeans, 67.9 kg of dark green leafy vegetables, 16.7 kg of vitamin A source other vegetables, 61.2 kg of vitamin C rich vegetables, 14.4 kg of other vegetables, 262.7 kg of other fruits, 3.4 kg of small fish with bones, 64.6 kg of eggs, 14.5 kg of fortified vegetable oil and 22.3 kg of fortified milk powder (see for details Supplementary material 2). As the dietary intake data was collected during the hunger season, we can assume that this optimised diet is also realistic for the rest of the year given that it is the more ‘challenging’ season.

### Farm size

The average farm size reported by the household in the dietary intake study was 2.1 ha. The majority of the households had a farm size below 3 ha (65% of the households), with 45% of the households below 2 ha and 17% below 1 ha (see Supplementary material 3 for the frequency distribution of the reported farm size).

### Crop availability and market information

We used secondary data sources for yields, waste factors, crop availability, crop land use and prices for all crops produced in Northern Ghana. We checked the data for plausibility with local experts.

#### Seasons

We divided the year into four seasons of three months based on the typical period of the dry season and the rainy/cropping season in Northern Ghana, combined with periods of food deficits: the first part of the dry season from November to January without food deficits (Season 1), the second part of the dry season from February to April with food deficits (Season 2), the first part of the rainy season from May to July with food deficits (Season 3), and the second part of the rainy season from August to October without food deficits (Season 4).

#### Crops cultivated

Crops cultivated in Northern Ghana and included in our analysis are based on: the recall of crop cultivation of households that participated in the dietary intake study, all foods consumed by the infants and young children in the dietary intake study, and the crops reported in Northern Ghana in the Ghana Panel Survey (GPS) carried out from 2009–2010 (Institute of Statistical Social and Economic Research (University of Ghana) & Economic Growth Center (Yale University), 2009). We excluded foods that are picked from the wild as they are not cultivated by farmers and information on their availability is missing. The following crops were produced and/or consumed in Northern Ghana: maize (*Zea mays* L.), millet (*Eleusine coracana* (L.) Gaertn. and *Pennisetum glaucum* (L.) R.Br.), sorghum (*Sorghum bicolor (*L.) Moench), rice (*Oryza sativa* L.), cassava (*Manihot esculenta* Crantz.), cocoyam (*Colocasia esculenta* (L.) Schott), plantain (*Musa* x *paradisiaca* L.), sweet potato (*Ipmoea batatas* (L.) Poir), yam (*Dioscorea* spp.), cowpea (*Vigna unguiculata* (L.) Verdc.), pigeonpea (*Cajanus cajan* (L.) Millsp.), cashew nut (*Anacardium occidentale* L.), groundnut (*Arachis hypogaea* L.), sesame seeds (*Sesamum indicum* L.), soybean (*Glycine max* (L.) Merr.), ayoyo leaves (*Corchorus olitorious* L.), bra leaves (*Hibiscus cannabinus* L.), amaranth (*Amaranthus cruentus* L.), okro (*Abelmoschus esculentus* (L.) Moench.), tomatoes (*Solanum lycopersicum* L.), onion leaves (*Allium cepa* L.), cucumber (*Cucumis sativus* L.), eggplant (*Solanum melongena* L.), onions (*Allium cepa* L.), yellow melon (*Cucumis melo* L.), watermelon (*Citrullus lanatus* Thunb.), melon seeds (neri), shea butter (*Vitellaria paradoxa* C.F.Gaertn.), orange (*Citrus sinensis* (L.) Osbeck.), mango (*Mangifera indica* L.), papaya (*Carica papaya* L.) and baobab (*Adansonia digitata* L.).

#### Yield

Average yields of all crops were based on secondary sources in the following order: average yields in Karaga district in 2006 from Ministry of Agriculture (MoFA) (SRID MoFA, 2006), average yields in Ghana in 2015 from MoFA (SRID MoFA, 2015) and average yields in Ghana in 2016 from FAOSTAT (FAO, 2016; Pinstrup-Andersen, 2014). If average yields for specific crops were missing, we used other sources or assumed yields from comparable crops. The assumed yields were already corrected for harvest losses. To assess the effects of different interventions we also used improved crop yields in our analyses. We included best attainable yields: the largest yields attained in field experiments in a specific area (Tittonell & Giller, 2013) for cowpea, groundnut and soybean in Northern Ghana (Kermah et al., 2017). For other crops we used sources in the following order: modelled rain fed crop yields (Global Yield Gap Atlas, 2018) and best attainable yield of a crop or a comparable crop in regions with comparable ago-ecological characteristics. These best attainable yields were corrected for the fact that most crop yields realized on farms begin to plateau when they reach about 80% of the attainable yields (Cassman et al., 2003; Lobell et al., 2009). In addition, we used yields 50% above the current average yields assuming these yields more realistic due to interventions at farm household scale. Supplementary material 4 presents the yields for the different crops. We did not find any data for best attainable vegetable yields.

#### Waste

Yields were corrected for waste as not all parts of a crop are consumed, based on the USDA national nutrient database for standard reference (USDA, 2016). In case waste factors were missing, the waste factor of a comparable crop was used.

#### Crop availability per season

Crop availability was based on data from the LSMS for Northern Ghana (Institute of Statistical Social and Economic Research (University of Ghana) & Economic Growth Center (Yale University), 2009). Each household reported for each crop the start and the end month of the cropping season, whether the crop was stored and the percentage lost during storage. In addition, we used the FAO cropping calendar for the Guinea savannah zone in Ghana for the length of the growth period per crop (FAO, 2018). We combined both information sources to determine in which seasons crops are available taking storage losses into account. Some crops can be cultivated twice a year. If data for a specific crop was missing, data of a comparable crop was used. We included interventions that can expand the availability of crops: irrigation (only for crops that were not available in specific seasons) and improved storage (considering locally feasible options such as drying of vegetable leaves). Data showed that vegetables and fruits were not available in the second part of the dry season (Season 2). In this season, also the least number of crops were on the land. In case of local storage methods and/or irrigation possibilities, the availability of vegetables and fruits could be expanded into the next season.

#### Duration of land use per cropping cycle

We used the same information sources as for crop availability to determine the duration and seasons of land use by a crop. For vegetables with a short cropping cycle of about half a season as defined in this study, we assumed that, considering land preparation, spreading of harvesting or other management issues, the cropping cycle covers a full season. Fruit trees, being perennials, occupy land year-round.

#### Interventions targeting crop availability

We modelled three main situations for crop availability. First, we modelled the ‘no interventions situation’ in which we used current average yields. Second, we modelled interventions that can expand crop availability. We included both improved storage practices and irrigation availability for vegetables and fruits that otherwise were not available in specific seasons. Third, we modelled any intervention that can improve crop yields such as using improved inputs or management practices. We used both the best attainable yields and increased yields by 50% of the average current yields for all crops in our model (for details see supplementary material 5).

#### Food prices per season

We used the data on food prices collected through the market survey of the dietary intake study in July 2014, the first part of the rainy season (Season 3) (de Jager et al., 2018). Prices fluctuate throughout the year. Therefore, we derived relative price fluctuations per month for sorghum, maize, millet, rice, cassava and yam in Tamale over the past 12 years to translate our price data to the other seasons. We used the relative price fluctuations of one of these specific foods for other foods with comparable availability throughout the year.

Supplementary material 5 shows for each crop the data used in this study: yield, waste factor, availability per season, duration of land use and prices.

### Testing farm designs and interventions for nutrient adequate diets

We applied linear programming (LP), using the software package General Algebraic Modelling System (GAMS), to test what farm designs and which agricultural interventions resulted in nutritious diets in all seasons. The optimised food needs for an average household per season were used to calculate the total needs of each food group. These food group needs were the main constraints included in the model. We calculated the minimum farm size needed to cover the food group needs per season of an average household in Northern Ghana for different scenarios:

Minimizing crop area per season:

$$\mathrm{Minimise}\ (Total\_Areas)\ [\mathrm{ha}]$$

where *Total_Area*_*s*_ is total farm size per season *s*.

We used the largest area required across the four seasons, which represents the minimum farm size to achieve a nutritious diet, as an upper farm size constraint in the subsequent calculations. We then maximized the revenue (and potentially affordability of nutritious diets) from farming, defined as the monetary value of crop produce sold minus the costs of foods purchased, for an average household in Northern Ghana:

Maximizing revenue:

$$\begin{aligned}& \mathrm{Maximise}\;(\mathrm{Revenue}=\sum\nolimits_c\ {Value\_total\_crop\_sold}_c \\ & \quad -\ Total\_cost\_food\_purchased)\lbrack\mathrm{GH}\kern2pt{|}\kern-5pt{\mathrm{C}}\rbrack\end{aligned}$$

where *Value_total_crop_sold*_*c*_ is total monetary value of sold produce of crop* c* and *Total_cost_food_purchased* is the total cost of foods purchased*.*

We assumed the cost of production to be zero as generally input use is limited and mainly family labour is used in the study area. The value of home produced and consumed foods is not included in the revenue, implying that the calculated revenue is available for other household needs than food. We calculated the maximum revenues for different farming interventions relative to the revenues without interventions (based on average crop yields), both in GH₵/year/household and in GH₵/year/ha.

**Fig. 1:**
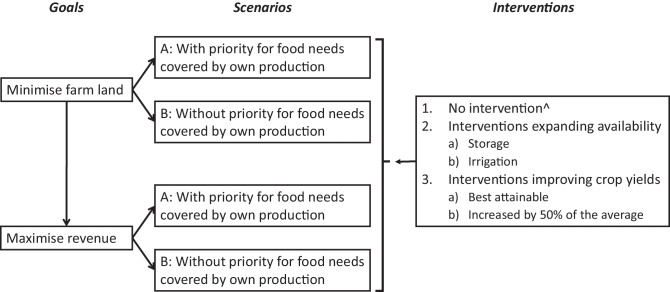
Goals, scenarios and interventions to cover a nutritious diet in all seasons of an average household in Northern Ghana. ^The situation of ‘no intervention’ is modelled by using the current average yields

We defined different scenarios for meeting a nutritious diet of a household. A scenario where all food group needs are covered by on-farm production, allowing only foods that could not be produced on farm (non-crop foods) or that are not available in specific seasons to be purchased. The costs for purchases are covered by crop sales. This scenario is further referred to as ‘with priority for food needs covered by own production'; focusing on own consumption (Scenario A). In the second scenario all food group needs are covered by on-farm production that can either be consumed or sold to purchase foods needed: no priority for either producing for own consumption or for selling. This scenario is further referred to as ‘without priority for food needs covered by own production' (Scenario B). We combined both scenarios separately with a range of different farm level interventions: (1) no intervention and average crop yields; (2) expanding availability of crops in food groups that could not be covered by own production based on (2a) storage and (2b) irrigation using average crop yields; (3) improved yields of grains, starchy crops, legumes and vegetables based on (3a) best attainable crop yields and (3b) yields increased by 50% of the average (see Fig. 1). The mathematical description of the models is included in Supplementary material 6.

## Results

### Scenario A: with priority to cover food needs by own production

#### Minimum farm size

**Fig. 2:**
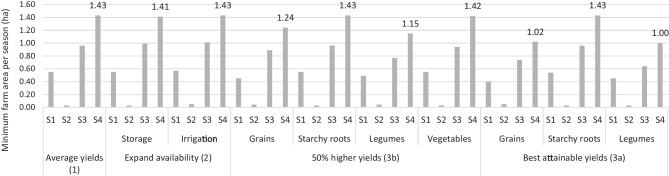
Minimum farm size needed for an average household in Northern Ghana to produce food that covers needs by own production (Scenario A). S1 = November to January (dry period), S2 = February to April (dry period), S3 = May to July (rainy period), S4 = August to October (rainy period). Expand availability = expand availability of vegetables and fruits. Storage = local feasible options such as drying of vegetable leaves but does not include cooling of fruits. Best attainable yields = the largest yields attained in field experiments in a specific area. 50% higher yields = yields 50% above the average yields. The numbers in brackets refer to the intervention (Fig. 1)

With average yields in scenario A, a total farm size of 1.43 ha is needed to produce food covering the dietary needs of an average household in Northern Ghana by own production (Fig. 2). For all selected interventions, the minimum farm size is determined by the area needed in the second part of the rainy season (Season 4). When increasing yields of different food groups (Intervention 3, Fig. 1), the minimum farm size needed for food production decreased. In case of a 50% yield increase (3b) or best attainable yields (3a) for grains 1.24 and 1.02 ha are needed, respectively, and for legumes 1.15 and 1.00 ha, respectively. A 50% yield increase of starchy crops did not influence the minimum farm size required. Increased yields of vegetables by 50% of their average, showed a minimal decrease in total farm size to 1.42 ha.

#### Maximum revenue

In this scenario, for each intervention the available farm size was set to the value obtained in Season 4 (Fig. 2), as explained in the previous section. Purchases of non-crop foods such as vegetable oil, fish, eggs, powdered milk, required a minimum amount of 5300 GH₵ per year. Additionally, as vegetables and fruits are not available in Season 2, they need to be purchased at an extra cost of 200 GH₵, except in case of expanding availability by storage (Fig. 1, Intervention 2a: 50 GH₵ extra) and irrigation (Intervention 2b: no extra costs). All available land will be cultivated in all seasons except for the second part of the dry season (Season 2) (Table 1) due to lack of water. Under irrigation (2b) also in Season 2 all land will be cultivated, resulting in the largest revenue, more than twice that of the standard average yields (Fig. 1, Intervention 1). Storage (2a) will not increase revenues. Only improving vegetable yields will substantially increase revenues (with 142% compared to average yields in GH₵/year/ha). Improving yields of grains, starchy roots and legumes will not increase revenue compared with standard average yields, but improving yields of grains and legumes will decrease land size needed and in case of legumes the revenue in GH₵/year/ha will remain similar to standard average yields (with 93% and 90%, respectively for best yields (3a) and 50% higher yields (3b)).

**Table 1:** Maximum revenue^ for an average household in Northern Ghana with priority to cover food needs by own production (scenario A)

	Farm size cultivated (ha)		Maximum revenue		Crops harvested			
	*S1, S3, S4*	*S2*	*GH₵/year*	*GH₵/year/ha*	*S1*	*S2*	*S3*	*S4*
1. Average yields	1.43	0.26	116600 (100%)	81538 (100%)	maize, rice, cowpea, groundnut, soybean, watermelon	sweet potatoes	bra leaves, amaranth, okro, onion, papaya	maize, cowpea, groundnut, soybean, bra leaves, okro, onion, watermelon
					*differences compared with ‘average yields’ scenario*			
2. Expand availability^a^								
a. Storage^b^	1.41	0.33	88%	90%	-	-	*ayoyo leave,* *no: bra leaves*	*oranges, no: bra leaves, okro, watermelon*
b. Irrigation	1.43	1.43	227%	227%	*ayoyo leaves, amaranth, okro, onion*	*onion, watermelon*	-	*no: bra leaves, okro, onion*
3. Improved crop yields								
a. Best yields^c^								
grains	1.02	0.38	54%	75%	*no: rice*	-	-	*oranges, no: watermelon*
starchy roots	1.43	0.26	111%	111%	-	-	-	-
legumes	1.00	0.22	65%	93%	-	-	-	*oranges*
b. 50% higher yields^d^								
grains	1.24	0.31	78%	90%	-	-	-	*oranges, no: watermelon*
starchy roots	1.43	0.26	102%	102%	-	-	-	-
legumes	1.15	0.28	72%	90%	-	-	-	*oranges, no: watermelon*
vegetables	1.42	0.31	141%	142%	-	-	-	*oranges, no: watermelon*

^Maximal revenue is the monetary value of own production minus the costs of foods purchased (5300 GH₵/year for non-crop foods plus additional 200 GH₵ for vegetables and fruits in Season 2 (5500 GH₵/year), except in case of expanding availability (for storage: 50 GH₵ extra (5350 GH₵/year), for irrigation: none (5300 GH₵/year))

S1 = November to January (dry period), S2 = February to April (dry period), S3 = May to July (rainy period), S4 =–August to October (rainy period)

- = no differences in crops harvested compared with ‘average yields’ scenario

^a^expand availability of vegetables and fruits

^b^storage includes local feasible options such as drying of vegetable leaves

^c^the largest yields attained in field experiments in a specific area

^d^yields 50% above the average yields

With all interventions a diversity of foods needs to be produced throughout the year including: maize, rice, cowpea, groundnut, soybean, watermelon, sweet potatoes, bra leaves, amaranth, okro, onion, papaya and watermelon (Table 1). In case of storage (2a) and irrigation of vegetables and fruits (2b), some other and/or additional dark green leafy vegetables, vitamin C rich vegetables and fruits need to be produced. Different farm sizes resulted in different crop combinations. The selection of crop combinations is driven by two factors. First, the model needed to fulfil the constraints to cover the food needs for an optimal diet by own crop production. Second, the goal of the model is to maximize revenue. These drivers together resulted in the selection of crops with largest yields per ha to fulfil the optimal diet constraints with a minimum land use and, for the remaining land, crops with the highest price per ha. In these scenarios farmers will produce most of their food needs themselves and therefore will have (almost) no costs for foods that need to be purchased.

### Scenario B: without priority to cover food needs by own production

#### Maximum revenue

The available land area for each intervention in Scenario B was maintained from Scenario A (areas in Season 4 in Fig. 2). In Scenario B with no priority to cover food needs by own crop production, for all interventions, all produce will be sold and all foods needed for a nutritious diet will be purchased from the revenues from crop production. The total costs of the optimal diet for the average household were 9900 GH₵/year (Table 2). The total farm size will be cultivated in all seasons for all interventions. In this scenario the crop combinations selected, are only driven by the goal of the model to maximise revenue and therefore crops were selected that yield the highest price per ha. For most interventions, sweet potatoes and onions need to be grown. Sweet potatoes need to be planted in the first part of the dry season (Season 1) and harvested in the second part (Season 2) and onions will be harvested both in first and second part of the rainy season (Season 3 and 4). Only in case of irrigation, onions will be harvested in each season. Therefore, this scenario resulted in the largest relative revenue of 185% compared to standard average yields in GH₵/year/ha. Improving vegetable yields will be, similar to results in scenario A, the most lucrative with 147% increased revenue in GH₵/year/ha. Only improving yield of starchy roots (best yields, 3a) will also increase revenue (127%) compared to standard average yield, but for none of the other crops improving their yields will result in larger revenues.

**Table 2:** Maximum revenue^ for an average household in Northern Ghana without priority to cover food needs by own production for consumption (scenario B)

	Farm size cultivated (ha)	Maximum revenue		Crops harvested			
	*S1, S2, S3, S4*	*GH₵/year*	*GH₵/year/ha*	*S1*	*S2*	*S3*	*S4*
1. Average yields	1.43	279400 (100%)	195385 (100%)	none	sweet potatoes	onion	onion
				*differences compared with ‘average yields’ scenario*			
2. Expand availability^a^							
a. Storage^b^	1.41	99%	100%	-	-	-	-
b. Irrigation	1.43	185%	185%	*onion*	*onion, no: sweet potatoes*	-	-
3. Improved crop yields							
a. Best yields^c^							
grains	1.02	70%	99%	-	-	-	-
starchy roots	1.43	127%	127%	-	-	-	-
legumes	1.00	69%	98%	-	-	-	-
b. 50% higher yields^d^							
grains	1.24	86%	99%	-	-	-	-
starchy roots	1.43	104%	104%	-	-	-	-
legumes	1.15	80%	99%	-	-	-	-
vegetables	1.42	146%	147%	-	-	-	-

^Maximum revenue is the monetary value of own production minus the costs of foods purchased. In all scenarios all food needs are purchased and the total minimum costs to purchase all

food needs are 9900 GH₵/year for an average household in Northern Ghana

S1 = season from November to January (dry period), S2 = season from February to April (dry period), S3 = season from May to July (rainy period), S4-season from August to October (rainy period)

- = no differences in crops harvested compared with ‘average yields’ scenario

^a^expand availability of vegetables and fruits as we found they cannot be harvested throughout the year

^b^storage includes local feasible options such as drying of vegetable leaves

^c^the largest yields attained in field experiments in a specific area

^d^yields 50% above the average yield

#### Minimum farm size

To cover food needs without a priority to cover food needs by own production (Scenario B), 0.10 ha cultivated with onions will be sufficient to earn 9900 GH₵/year. The intervention with improved yields for vegetables (3b; onions) showed that a farm size of 0.07 ha will be sufficient.

## Discussion

### Do the households have sufficient land?

Our model results suggest that the average farm size of households in rural Northern Ghana should be sufficient to produce a nutritious diet. Assuming average crop yields, a minimum farm size of 1.43 ha is needed to cover the food needed for a nutritious diet from own production. Households in the dietary intake study reported a median farm size of 2.1 ha with 75% of the households above 1.43 ha. A legume cultivation project (N2Africa) in the same region reported an average farm size of 2.8 ha (Franke et al., 2011). Therefore, farm size does not (yet) seem to be a limiting factor in rural Northern Ghana to produce the food needed for a nutritious diet. With the expected population growth (Population Reference Bureau, 2018) and the further division of farm land area by inheritance, it is expected that household land area will decrease in the future. For households with smaller farms, our study results indicate that increasing yields, especially of legumes and grains, is an option to enable households to cover their food needs for a nutritious diet. As increasing yield allows for a reduction of field size, this in turn would open up the possibility to free up space for the production of other foods belonging to a nutritious diet, such as vegetables. Snapp and Fisher (2015) found, for example, that agricultural subsidies to intensify maize production in Malawi were associated with greater crop diversity and household dietary diversity suggesting that the adoption of modern varieties of maize may indeed 'have freed up farmers to grow more mixed crops'. However, in general, interventions increasing yield will also increase the cost of inputs. As households with smaller farm sizes also tend to be poorer in terms of total value of household assets per household member (positive correlation in the dietary intake study, r = 0.81, n = 329, P-value = 0.00), this may limit the success of yield increasing interventions.

### Seasonality

Our findings confirm that household vegetable and fruit dietary needs cannot be covered by home production during the second part of the dry season, the so-called hunger season, unless irrigation is available. In general, in rural settings in LMICs, food availability indeed varies between seasons and access to perishable but often nutrient-dense foods such as fruits and vegetables can be limited (HLPE, 2017). During the hunger season, food availability and accessibility are often inadequate, as stored supplies are exhausted and demands are high, leading to high food prices on the market (Devereux, 2009) and reduced affordability. Masters et al. (2018) reported that the costs of a diverse diet in Ghana fluctuated throughout the seasons as was also reflected in our price data. Another study in Northern Ghana found a less diverse diet among school children during the end of the dry season compared with the end of the growing season, especially less vitamin A-rich fruits and vegetables were consumed during the dry season (Abizari et al., 2017). Seasonal variations in the consumption of fruits, legumes, roots and plantains were also reported among pre-school children in Ghana (Ferguson et al., 1993). In addition, diseases are more prevalent and labour demands are strongest at the start of the rainy season, which both further increase the demand for foods to cover increased nutrient and energy requirements in the period when least food is available (Devereux, 2009), especially of perishable foods such as vegetables and fruits. Expanding availability of vegetables and fruits by irrigation of vegetables and some fruits (watermelon), can cover the needs of all food crops of the household through their own farm production. Rice and vegetables dominate the small irrigated crop sector in Ghana, with 50% of vegetable production being irrigated, often in combination with rice on the same fields (FAO, 2014). Effective irrigation techniques such as treadle and solar pumps may close food gaps in the hunger season. A review of the linkages between irrigation, food security and nutrition indeed concluded that irrigation contributed to improving food security but there is no evidence of impacts on nutrition due to a lack of studies that included nutrition outcomes (Domènech, 2015). Expanding availability of vegetables, for example by drying of vegetable leaves for consumption during the hunger season, were only able to partly close the vegetable gaps in our models. Generally, the consumption of vegetables remains insufficient and studies agree that Africa has the lowest fruit and vegetable consumption (Kalmpourtzidou et al., 2020; Micha et al., 2015). The insufficient consumption combined with the challenges of vegetable availability, emphasises the importance that besides the availability of vegetables, also the accessibility, affordability and desirability need to be addressed (GLOPAN, 2020).

### Diversification of cropping systems

The model results suggest that households need to produce a diversity of foods to cover their dietary needs from their own production (scenario A). For all interventions and achieving minimal farm size needs, the following locally available foods need to be produced in different amounts to cover the needs for a nutritious diet: maize, rice, cowpea, groundnut, soybean, watermelon, sweet potatoes, bra leaves, amaranth, okro, onion, papaya and watermelon. However, an earlier study in the same area found that 60% of the households did not produce enough grains and legumes and none of the households produced sufficient vegetables to cover their needs on a yearly basis (de Jager et al., 2018). Model results indicate that households need to grow a wide variety of crops for their own food provisioning. For farming households that consume their own produce complemented with food bought on the local market, on-farm diversity is related to dietary diversity. However, as soon as the local market receives food from other areas, the link between on-farm diversity and diet diversity weakens (Moges et al., 2022; Remans et al., 2011; Sibhatu et al., 2015). Our study results also suggest that only increasing farm diversity by smallholders will not close nutrient gaps in itself, as many are limited by their farm size to be able to produce all their nutrient needs and not all farmers will achieve the average yields. Furthermore, it may be difficult to adapt their crop rotations due to labour constraints (Nin-Pratt & McBride, 2014), seasonality and knowledge about the cultivation of specific crops.

Our model results also suggest that when households do not need to produce their own food needs (scenario B), producing one or two of the most lucrative cash crops and purchasing all their food needs will result in the highest revenue. Although specialisation in the most profitable crop is a short-term economic option to increase income of rural households (Klasen et al., 2016; Sibhatu & Qaim, 2018), small farms will rarely produce only one or two crops in order to avoid the risks related to diseases, weather and market shocks. In addition, due to inelastic food markets the scenario of producing only one or two profitable crops is unrealistic as the market will become saturated when supplied by many households. Furthermore, markets and infrastructure need to function well: all of the cash crops need to be sold and sufficient diverse foods need to be available and affordable at the market at the right time. This does not mean that self-sufficiency should necessarily be promoted over commercialization, but it shows the fact that dysfunction in agricultural markets is hampering the natural transformation towards a more commercially-oriented sector (Ecker, 2018). In addition to the need of well-functioning markets, the income also needs to be used to purchase the quantities and diversity of foods needed to cover the food and nutrient needs of a household, an assumption that rarely holds (Herforth & Ahmed, 2015; Jones, 2017). As production and consumption decisions are not separable and based on market signals and perceived risks, factors influencing household decisions need to be taken into account when ultimately recommending strategies (Ecker, 2018). To ensure that mainstream agricultural interventions will result in nutritious diets, all key elements of the food environment need to be aligned (GLOPAN, 2020).

### Potential agricultural interventions

Among the mainstream agricultural interventions tested and compared with average yields, irrigation and increasing yield of vegetables resulted in the relative highest revenue in both scenarios A and B. With irrigation, crop cultivation can be extended to more seasons also including the opportunity of extra vegetables (our findings show as being most lucrative) to be cultivated. Although irrigation scenarios resulted in a doubled relative revenue compared to standard yields, the costs of irrigation are not included in our model and will probably significantly reduce the relative revenue. Due to the initial investment required for irrigation, it is unlikely to be a feasible option for poorer households. The increased vegetable yields scenario, includes cultivation of onion, watermelon and sweet potato suggesting these to be the most lucrative in Northern Ghana, as agreed by local experts. Increasing yields of grains, starchy roots and legumes did not increase revenue compared with standard average yields. But increasing yields of grains and legumes did decrease land size needed while, especially for legumes, resulting in similar revenues as standard average yield scenario. This implies that increasing yields of legumes and grains, provided they can improve management and/or afford inputs, will allow households with a limited farm size to maintain a similar level of revenue while covering their food needs for a nutritious diet.

This study shows that taking a nutritious diet as a starting point provides valuable insights into diversification of the cropping system and the potential contribution of agricultural interventions to achieving nutritious diets throughout the year, either via increased food availability and/or affordability. Although the average farm size in rural Northern Ghana should be sufficient to produce a nutritious diet, seasonality overrides. Unless irrigation is available, the household’s vegetable and fruit dietary needs cannot be covered during the hunger season. Increasing yields of legumes and cereal grains would allow households with a limited farm size to maintain a similar level of revenue while covering their food needs for a nutritious diet. When households do not produce enough food and need to generate income to purchase food, specialisation in cash crop production is attractive. Yet specialisation comes with increased risks related to diseases, weather and market shocks.

Overall, our findings confirm what is found and/or suggested in other studies. In addition, the study shows more specifically how and in what range different interventions can contribute to nutritious diets. Furthermore, this study illustrates how food-based dietary guidelines (FBDGs) can be used for agricultural investments that aim for supporting healthy diets. FBDGs are often seen as niche instruments for the health sector and used in behaviour change communication to inform consumers on what (not) to eat. It is often highlighted that FBDGs should also be used as providing dietary standards or targets to programmes in other (public and private) sectors such as agriculture. We used FBDGs to identify what crops to cultivate to fulfil nutrient adequacy. Appropriate FBDGs are based on the actual dietary patterns of a specific population, and therefore represent a locally acceptable nutritious diet based on available and acceptable foods (Ferguson et al., 2006). FBDGs make ‘reverse thinking’ possible by starting with a healthy diet perspective to identify food environment characteristics, and addressing these in innovations aiming at healthier diets (Brouwer et al., 2021).

### Scope of the study

Modelling different scenarios provided useful insights on potential possibilities and limitations of complex situations, taking into account different aspects simultaneously. As the model results largely depend on the data used and assumptions made, their implications for the model outcomes need to be acknowledged. First, we were limited by the availability of primary and secondary data, especially of seasonally-specific data of crop yields, crop availability and crop prices. For crop availability throughout the year no data were available at all as seasonality in relation to agricultural activities, food availability and a nutritious diet is rarely studied in detail. Second, we had to make assumptions for those missing data which may have influenced our model results. For example, with regard to the crop yield data, we assumed that the yields as reported by the Ministry of Agriculture excluded harvest loss. If this was not the case, we have overestimated the actual yields and underestimated the minimum farm size. Besides harvest loss, many other factors may contribute not achieving the assumed yield such as accessibility to quality seeds, fertilization, knowledge and markets. With regard to the seasonal food prices, we used monthly price data for sorghum, maize, millet, rice and cassava and assumed similar fluctuations throughout the year for other crops. However, for vegetables the fluctuations may have been more extreme as they are perishable and not available when produced without irrigation in dry seasons (IFPRI, 2020). Unfortunately, price data for specific seasons and markets closest to study location were not available. Further we assumed labour and input costs to be negligible as generally mainly family labour is used and input use is low in our study area. Nevertheless, whenever family labour is used this means this labour potential cannot be used to earn an income elsewhere. Labour is reported to be a major constraint in Ghana (Nin-Pratt & McBride, 2014). Inputs are needed to get best attainable yields and possibly also for a 50% yield increase. In addition, other production costs such as upfront investments in technologies as irrigation systems, are also not included in the study. Therefore, we probably overestimated the land that actually can be cultivated in all seasons given labour constraints, as well as overestimating maximum revenue as costs of production are not accounted for and market prices are used as sales prices, neglecting price differences related to presence of middlemen. Hence, we cannot draw conclusions with regard to the absolute revenues calculated. However, the scenarios and interventions are assessed consistently, based on the available data and literature, and hence, we trust that the relative differences among scenarios reflect reality. We reported relative revenues for all scenarios compared with the standard average yield. We checked the sensitivity of the models to prices and found that the relative revenues are not affected (Supplementary material 7) and thus comparison between scenarios is considered valid. Third, the calculated minimal costs for our optimal diet of 9900 GH₵ per year for an average household of 14 members are within the range of the reported food expenditures in the LSMS (Ghana Statistical Service, 2014) but at the lower end of the distribution. This corresponds with our minimized costs for the optimal diet. Another study that calculated the price of an optimal diet in Ghana, reported a cost of 4.68 GH₵ per person (Anker, 2006), comparable to the costs of our modelled optimised diet in Optifood of 4.30 GH₵ per person. Fourth, we did not include livestock rearing in the model which may also contribute to the availability of animal-sourced foods in a household. The optimal diet included only eggs as animal-sourced foods that also may be provided by livestock rearing but, in our model, we assumed eggs were purchased. In Northern Ghana, small-scale livestock rearing serves mostly as a safety net to quickly access cash for emergency (medical) or planned expenditures (school fees) (Roelen, 2017). As these are non-food expenditures and only few animal-sourced foods were included in our optimal diet, excluding livestock is assumed to have limited effect on our results with regard to covering the food needs. However, with regard to the effect on our revenue, results depend on how much a household can earn from livestock rearing and how much land is needed for feeding the animals.

### Conclusion

Our study shows the value of modelling the range of dietary effects from agricultural interventions in a specific context, using a local feasible nutritious diet as a starting point and taking seasonality into account. Our modelling shows that ensuring a year-round nutritious diet requires enhanced availability of vegetables and fruits in the hunger season in rural Northern Ghana. Although staple crops do not provide the full range of essential nutrients, increasing their yields allows for a reduction of field size freeing up space for the production of other foods belonging to a nutritious diet, such as vegetables. Nevertheless, small farms (less than 1.4 ha) are unable to produce sufficient food to cover their needs. They will depend on their income both from agriculture and other sources, and on the availability of types of foods on markets to meet their dietary needs. Our study results suggest that interventions are needed to ensure fruit and vegetable availability and affordability year-round.

## Supplementary Information

Below is the link to the electronic supplementary material.

Supplementary file1 (DOCX 131 KB)

## Data Availability

All data supporting the findings of this study are available from the corresponding author on request.

## Code availability

Commands and codes used for analysis in General Algebraic Modelling System (GAMS) are available from the corresponding author on request.
