# Supplementary material for: Seasonality and nutrition-sensitive farming in rural Northern Ghana
Source: Food Secur. 2022 Nov 23;15(2):381–94. doi: 10.1007/s12571-022-01325-5 (PMC10066165; doi:10.1007/s12571-022-01325-5)
Supplement: Supplementary file 1 — Supplementary file1 (DOCX 131 KB) [file 12571_2022_1325_MOESM1_ESM.docx]

# Supplementary materials

For the manuscript titled: Seasonality and nutrition-sensitive farming in rural Northern Ghana
Ilse de Jager^*^, Gerrie W.J. van de Ven, Ken E. Giller, and Inge D. Brouwer

^*^Corresponding author: I. de Jager, Division of Human Nutrition and Health, Wageningen University, P.O. Box 17, 6700 AA Wageningen, The Netherlands. Email: [ilsedejager@wur.nl](mailto:ilsedejager@wur.nl)

## Supplementary material 1

### Method description for retrieving the optimal nutrient adequate diet for an average household

Based on the household information and the respective estimated average requirements (EARs) of 11 key micronutrients, we calculated the number of consumer units in a household relative to women 19-50 years not pregnant or lactating (denoted one consumer unit), see Table 1 for details. A child of 12-23 months was determined at 0.5 consumer units. The food needs per consumer unit were based on the optimized diet for non-breast children of 12-23 months multiplied by two. In our study region the diet of children older than one year is integrated in the family diet (GAIN, 2016). An average household in Karaga district consisted of 12.2 consumer units (Table 2). This number was multiplied by the optimised food needs per consumer unit to arrive at total household food needs in g per day and in kg per season of 3 months (see below for the definition of a season). This translation of the results of Optifood to an average household ensured that nutrient needs of all household members were approximately met by this diet. The diet covered all nutrients above 70% of the summed recommended nutrient intake (RNI) of an average household, except for fat for which 53% was covered (Table 3). For most nutrients an intake above 70% of the RNI represents at least the EAR. The 70% cut-off is also used by others allowing for comparison (Kujinga et al., 2018; Santika et al., 2009; Talsma et al., 2018). We set the minimum fat intake at 30% of total energy intake while the required range is 20 to 35% (FAO, 2010), therefore the coverage of fat needs is still above the lower boundary of the adequate range.

### Table 1

**Consumer units (CU) for translation of modelled optimised diet for non-breastfed children 12 to 23months old to optimised diet for an average household**

| **Groups WHO** | **Calcium** | **Zinc** | **Iron** | **Vitamin A** | **Thiamine** | **Riboflavin** | **Niacin** | **Vitamin B6** | **Folate** | **Vitamin B12** | **Vitamin C** | ***Average*** | ***Average, merged groups**** |
| --- | --- | --- | --- | --- | --- | --- | --- | --- | --- | --- | --- | --- | --- |
|  | *Consumer Units (CU)* | | | | | | | | | | | |  |
| 0 - 6 months | 0.1 | **0.8** | 0 | 0 | 0.1 | 0.1 | 0.1 | 0 | 0 | 0 | 0 | *0.4* | *0.5* |
| 7 - 12 months | 0.1 | 0.5 | 0.5 | 0 | 0.2 | 0.2 | 0.3 | 0.2 | 0 | 0 | **0.7** | *0.5* |  |
| 1 - 3 y | 0.3 | 0.2 | 0.4 | 0 | 0.3 | 0.2 | 0.4 | 0.3 | 0.2 | 0.1 | 0.1 | *0.5* | *0.7* |
| 4 - 6 y | 0.6 | 0.6 | 0.5 | 0.7 | 0.5 | 0.5 | 0.6 | 0.4 | 0.5 | 0.5 | 0.7 | *0.6* |  |
| 7 - 9 y | 0.7 | 0.6 | 0.5 | 0.9 | 0.8 | 0.8 | 0.9 | 0.7 | 0.8 | 0.8 | 0.8 | *0.7* |  |
| Females, 10 - 14 y, pre-menarche | 1.3 | 1.0 | **0.6** | **1.4** | 1.0 | 1.0 | 1.1 | 0.9 | 1.0 | 1.0 | 1.0 | *1.0* | *1.1* |
| Females, 10 - 14 y, menarche | 1.3 | 1.0 | 0.7 | **1.4** | 1.0 | 1.0 | 1.1 | 0.9 | 1.0 | 1.0 | 1.0 | *1.0* |  |
| Females, 15 - 18 y | 1.3 | 1.3 | 1.0 | 1.4 | 1.0 | 1.0 | 1.1 | 0.9 | 1.0 | 1.0 | 1.0 | *1.1* |  |
| Males, 10 - 14 y | 1.3 | 1.0 | **0.7** | 1.4 | 1.2 | 1.1 | 1.1 | 1.0 | 1.0 | 1.0 | 1.0 | *1.1* | *1.1* |
| Males, 15 - 18 y | 1.3 | **1.6** | 0.9 | 1.4 | 1.2 | 1.1 | 1.1 | 1.0 | 1.0 | 1.0 | 1.0 | *1.1* |  |
| Females, 19 - 50 y, premenopausal | 1.0 | 1.0 | 1.0 | 1.0 | 1.0 | 1.0 | 1.0 | 1.0 | 1.0 | 1.0 | 1.0 | *1.0* | *1.0* |
| Females, 51 - 65 y, menopausal | 1.3 | 1.0 | **0.5** | 1.0 | 1.0 | 1.0 | 1.0 | 1.2 | 1.0 | 1.0 | 1.0 | *1.0* |  |
| Females, 65+ y | 1.3 | 1.0 | **0.5** | 1.1 | 1.0 | 1.0 | 1.0 | 1.2 | 1.0 | 1.0 | 1.1 | *1.0* |  |
| Pregnant women | **1.2** | 1.7 | **3.8** | 1.4 | 1.3 | 1.3 | 1.3 | 1.5 | 1.6 | **1.1** | 1.3 | *1.6* | *1.6* |
| Lactating women | 1.0 | 1.3 | **0.7** | **1.7** | 1.5 | 1.4 | 1.2 | 1.5 | 1.4 | 1.2 | **1.7** | *1.3* | *1.3* |
| Males, 19 - 50 y | 1.0 | **2.1** | **0.7** | 1.1 | 1.2 | 1.1 | 1.1 | 1.0 | 1.0 | 1.0 | 1.1 | *1.1* | *1.2* |
| Males, 51 - 65 y | 1.0 | **2.1** | **0.7** | 1.1 | 1.2 | 1.1 | 1.1 | 1.3 | 1.0 | 1.0 | 1.1 | *1.2* |  |
| Males, 65+ y | 1.3 | **2.1** | **0.7** | 1.1 | 1.2 | 1.1 | 1.1 | 1.3 | 1.0 | 1.0 | 1.1 | *1.2* |  |

Consumer units are based on EARs of each nutrient (WHO/FAO 2004) for specific group relative to EAR of women 19 to 50 years old who are not pregnant or lactating. Averages of the consumer units of all 11 nutrients were calculated for each group (average are calculated for each row). Average consumer units are used to calculate quantity of foods needed for each household member based on optimised diet for non-breastfed children 12 to 23 months old.
***Bold*** *= 0.4 or more CU difference of a specific nutrient with average CU for all nutrients for a specific group.*
***For the calculation of the total consumer units needed for an average household**: we used the median number of household members for different groups (infants of 0-12 months, children of 1-9 years, females of 10-18 years, males of 10-18 years, females ≥18 years, pregnant women, lactating women and males ≥19 years) and their respective calculated consumer unit. When within a specific age group there are different consumer units (different age categories within one group), the highest consumer unit was used.

### Table 2

**Average household composition (n=337) for each sex and age group^a^ and for the whole household: median number, consumer unit for specific group and consumer unit for average household**

| **Groups** | **Median [25^th^, 75^th^]** | **CU^b^ group** | **CU^b^**  **average household** |
| --- | --- | --- | --- |
| Infants, 0-12 mo | 1 [0, 1] | 0.5 | 0.5 |
| Children, 1-9 y | 5 [3, 7] | 0.7 | 3.5 |
| Females, 10-18 y | 1 [0, 2] | 1.1 | 1.1 |
| Males, 10-18 y | 1 [0, 2] | 1.1 | 1.1 |
| Females, >19 y | 3 [2, 6] | 1.0 | 1.0 |
| *Pregnant women, >19 y* | *0 [0, 1]* | *1.6* | *0* |
| *Lactating women, >19 y* | *2 [1, 2]* | *1.3* | *2.6* |
| Males, >19 y | 2 [1, 4] | 1.2 | 2.4 |
| Total household members | 14 [9, 21] |  | 12.2 |

^a^Groups are based on the groups as defined by (WHO & FAO, 2004) for EARs of nutrients. ^b^CU=consumer unit (for calculation see Table 1).

### Table 3

**Coverage of energy and nutrient needs by optimised diet for non-breastfed children of 12-23 months old (optimised diet used to translate to household food needs) and an average household**

|  | Children 12-23 months, non-breastfed (0.5 CU) | Average household (12.2 CU) |
| --- | --- | --- |
| Nutrients | Coverage %, RNI | |
| Energy (Kcal)^a^ | 107.4 | 70 |
| *Macronutrients* |  |  |
| Fat^b^ | 304.8 | **53** |
| Protein^c^ | 125.4 | 157 |
| *Micronutrients* |  |  |
| Calcium | **33.2** | 81 |
| Iron | 78.0 | **67** |
| Zinc | 150.7 | 104 |
| Vitamin A^d^ | **30.2** | 104 |
| Thiamin | 142.7 | 149 |
| Riboflavin | 98.6 | 131 |
| Niacin | 168.4 | 166 |
| Vitamin B6 | 153.1 | 102 |
| Folate | 89.4 | **69** |
| Vitamin B12 | **2.3** | 76 |
| Vitamin C | **42.1** | 110 |

Bold = coverage below 70% of RNI. The nutrient needs of an average household are calculated by the sum of the nutrient needs of the median number of persons for each specific age and/or sex group, selecting the person with the highest energy needs within the group assuming he/she also has the highest needs of other nutrients. ^a^energy requirements WHO 2001, assume moderate activity ^b^WHO 2010 AMDR, based on energy requirements
^c^Safe level.
^d^Recommended safe intakes.

## Supplementary material 2

#### Food and food group needs of the optimised diet for children not breastfed of 12-23 months and for an average household

|  | **Children not breastfed 12-23 months (0.5 CU)** | | | **Average household (12.2 CU)** | |
| --- | --- | --- | --- | --- | --- |
| **Foods per food group** | **Servings/week** | **Median serving size (g)** | **Quantity g/day** | **Quantity g/day** | **Quantity**  **kg/season^a^** |
| *Grains* |  |  |  | *2257* | *206.0* |
| Maize dough, white | 1 | 38.4 | 5.5 | 134 |  |
| Maize flour, white | 3 | 125 | 53.6 | 1305 |  |
| Maize grain, dried white oiled | 1 | 11 | 1.6 | 37 |  |
| Millet dough | 2 | 53.2 | 15.2 | 366 |  |
| Millet flour | 1 | 18.8 | 2.7 | 61 |  |
| Rice brown, unpolished | 1 | 102.7 | 14.7 | 354 |  |
| *Starchy foods* |  |  |  | *232* | *21.2* |
| Cassava flour | 2.5 | 26.7 | 9.5 | 232 |  |
| *Beans, lentils, peas* |  |  |  | *1013* | *92.4* |
| Cowpea, white dried | 4 | 41.6 | 23.8 | 586 |  |
| Pigeon peas, dried | 3 | 40.8 | 17.5 | 427 |  |
| *Nuts, seeds* |  |  |  | *756* | *69.0* |
| Groundnut roasted, paste | 7 | 25.2 | 25.2 | 610 |  |
| Melon seeds, roasted | 3 | 14.5 | 6.2 | 146 |  |
| *Soybeans and products* |  |  |  | *158* | *14.4* |
| Soybeans, dried | 4 | 1.8 | 1.0 | 24 |  |
| Soybeans flour | 3 | 13.2 | 5.7 | 134 |  |
| *Dark green leafy vegetables* |  |  |  | *744* | *67.9* |
| Ayoyo (jute) leaves | 4 | 18.3 | 10.5 | 256 |  |
| Baobab leaves, dried | 1 | 8.2 | 1.2 | 24 |  |
| Bra (kenaf) leaves | 7 | 18.9 | 18.9 | 464 |  |
| *Vitamin A source other vegetables* |  |  |  | *183* | *16.7* |
| Tomato paste | 5 | 9.7 | 6.9 | 171 |  |
| Tomato powder, dried | 2 | 1.7 | 0.5 | 12 |  |
| *Vitamin C-rich vegetables* |  |  |  | *671* | *61.2* |
| Okro fruit | 7 | 27.5 | 27.5 | 671 |  |
| *Other vegetables* |  |  |  | *158* | *14.4* |
| Onion bulb | 7 | 5.7 | 5.7 | 134 |  |
| Okro fruit powder, dried | 2 | 4.2 | 1.2 | 24 |  |
| *Other fruits* |  |  |  | *2879* | *262.7* |
| Watermelon | 7 | 117.9 | 117.9 | 2879 |  |
| *Fish, small with bones* |  |  |  | *37* | *3.4* |
| Mackerel canned in tomato sauce | 7 | 1.3 | 1.3 | 37 |  |
| *Eggs* |  |  |  | *708* | *64.6* |
| Egg guinea fowl | 7 | 28.8 | 28.8 | 708 |  |
| *Vegetable oil, fortified* |  |  |  | 159 | 14.5 |
| Oil vegetable Frytol | 7 | 6.4 | 6.4 | 159 |  |
| *Fluid/powdered milk, fortified* |  |  |  | *244* | *22.3* |
| Milk powder, cow skimmed | 7 | 10.0 | 10.0 | 244 |  |

^a^Recommended quantity per season of 3 months (91.25 days)

## Supplementary material 3


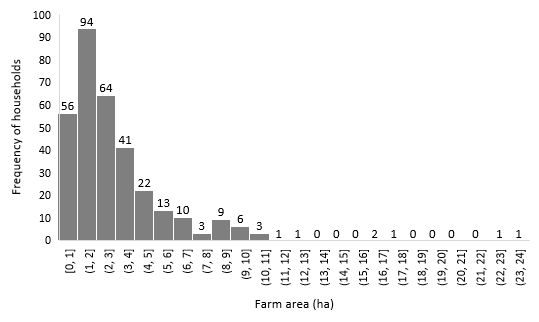

*Frequency of households per range of farm size (n=329, 1 excluded in figure with farm size of 45 ha)*

## Supplementary material 4

**Crops produced in Northern Ghana: average yields, average yields increased by 50%, and best attainable yields**

| **Crops cultivated per food group** | **Average yield**  **(t ha^-1^)** | **Average yield + 50% (t ha^-1^)** | **Best attainable**  **yield^a^ (t ha^-1^)** |
| --- | --- | --- | --- |
| *Grains* |  |  |  |
| Maize | 1.6 | 2.3 | 5.9 |
| Millet | 0.8 | 1.2 | 2.6 |
| Sorghum | 0.9 | 1.4 | 5.5 |
| Rice | 1.5 | 2.3 | 4.7 |
| *Starchy foods* |  |  |  |
| Cassava | 10.0 | 15.0 | 40.0 |
| Cocoyam | 10.0 | 15.0 | 40.0 |
| Plantain | 10.9 | 16.4 | 40.0 |
| Sweet potatoes | 10.0 | 15.0 | 40.0 |
| Yam | 9.4 | 14.1 | 40.0 |
| *Beans, lentils, peas* |  |  |  |
| Cowpea | 1.2 | 1.8 | 2.3 |
| Pigeon peas | 1.2 | 1.8 | 2.2 |
| *Nuts, seeds* |  |  |  |
| Cashew nuts | 0.6 | - | - |
| Groundnut | 0.7 | 1.1 | 1.4 |
| Melon seeds | 0.1 | - | - |
| Sesame seeds | 0.1 | - | - |
| *Soybeans and products* |  |  |  |
| Soybeans | 0.8 | 1.2 | 4.5 |
| *Dark green leafy vegetables* |  |  |  |
| Ayoyo (jute) leaves | 9.0 | 13.5 | - |
| Bra (kenaf) leaves | 9.0 | 13.5 | - |
| Amaranth | 9.0 | 13.5 | - |
| *Vitamin C rich vegetables* |  |  |  |
| Okro | 23.2 | 34.7 | - |
| Tomatoes | 8.3 | 12.5 | - |
| Onion leaves | 9.2 | 13.8 | - |
| *Other vegetables* |  |  |  |
| Cucumber | 13.8 | 20.7 | - |
| Onion | 18.8 | 28.2 | - |
| Eggplant | 8.9 | 13.3 | - |
| *Other fruits* |  |  |  |
| Yellow melon | 15.4 | - | - |
| Watermelon | 26.1 | - | - |
| Shea fruit | 0.8 | - | - |
| Orange | 19.9 | - | - |
| Mango | 7.3 | - | - |
| Papaya | 19.4 | - | - |

*- = no best yield available/not modelled* ^a^the largest yields attained in field experiments in a specific area

## Supplementary material 5

**Descriptive data used in the model**

*s* = season (1 to 4)

| s1 | Nov_Jan_dry1 |
| --- | --- |
| s2 | Feb_April_dry2 |
| s3 | May-July_rain1 |
| s4 | Aug_Oct_rain2 |

*f* = food group (1 to 14)

| ***f*** | **Food group description** | ***f* requirement**  *average hh in kg/s* | ***f* availability on farm per s**  *o=no, 1=yes* | | | | | |
| --- | --- | --- | --- | --- | --- | --- | --- | --- |
|  |  |  | s1 | s2 | *s2^a^* | *s2^b^* | s3 | s4 |
| 1 | vegetable_oil | 14.51 | 0 | 0 | *0* | *0* | 0 | 0 |
| 2 | whole_grains_and_products | 205.95 | 1 | 1 | *1* | *1* | 1 | 1 |
| 3 | cooked_beans | 92.44 | 1 | 1 | *1* | *1* | 1 | 1 |
| 4 | nuts_and_seeds | 68.99 | 1 | 1 | *1* | *1* | 1 | 1 |
| 5 | soybeans_and_products | 14.42 | 1 | 1 | *1* | *1* | 1 | 1 |
| 6 | starchy_plant_foods | 21.17 | 1 | 1 | *1* | *1* | 1 | 1 |
| 7 | small_fish_with_bones | 3.38 | 0 | 0 | *0* | *0* | 0 | 0 |
| 8 | eggs | 64.61 | 0 | 0 | *0* | *0* | 0 | 0 |
| 9 | vitamin_A_source_darkgreenleafyvegetables | 67.89 | 1 | 0 | *1* | *1* | 1 | 1 |
| 10 | vitamin_A_source_other_vegetables | 16.70 | 0 | 0 | *0* | *0* | 0 | 0 |
| 11 | vitamin_C_rich_vegetables | 61.23 | 1 | 0 | *1* | *1* | 1 | 1 |
| 12 | other_vegetables | 14.42 | 1 | 0 | *0* | *1* | 1 | 1 |
| 13 | other_fruit | 262.71 | 1 | 0 | *0* | *1* | 1 | 1 |
| 14 | fluid_powdered_milk_fortified | 22.27 | 0 | 0 | *0* | *0* | 0 | 0 |

*^a^Availability in second season for storage scenario.
^b^Availability in second season for irrigation scenario.*

*c* = crops (names are used in modelling)

| **crop_name** | ***f*** | **Yield**  *Kg/ha* | **Yield^a^**  *Kg/ha* | **Yield^b^**  *Kg/ha* | **Yield^c^**  *Kg/ha* | **Waste**  *factor* | **Crop availability^d^** *(harvest+storage loss)* | | | | **Land needed**  *o=no, 1=yes* | | | | **Food prices**  *GH₵/kg* | | | |
| --- | --- | --- | --- | --- | --- | --- | --- | --- | --- | --- | --- | --- | --- | --- | --- | --- | --- | --- |
|  |  |  |  |  |  |  | s1 | s2 | s3 | s4 | s1 | s2 | s3 | s4 | s1 | s2 | s3 | s4 |
| sorghum_guineacorn | 2 | 930 | 930 | **5520** | **1395** | 1.00 | 1.00 | 0.88 | 0.88 | 0.88 | 1 | 0 | 1 | 1 | 3.85 | 3.28 | 3.60 | 4.07 |
| maize_1 | 2 | 1560 | 1560 | **5920** | **2340** | 1.00 | 0.90 | 0.90 | 0.00 | 1.00 | 0 | 0 | 1 | 1 | 2.12 | 2.00 | 2.30 | 2.23 |
| maize_2 | 2 | 1560 | 1560 | **5920** | **2340** | 1.00 | 1.00 | 0.90 | 0.90 | 0.00 | 1 | 0 | 0 | 1 | 2.12 | 2.00 | 2.30 | 2.23 |
| millet_1 | 2 | 770 | 770 | **2560** | **1155** | 1.00 | 0.88 | 0.88 | 0.00 | 1.00 | 0 | 0 | 1 | 1 | 5.06 | 4.05 | 4.40 | 5.10 |
| millet_2 | 2 | 770 | 770 | **2560** | **1155** | 1.00 | 1.00 | 0.88 | 0.88 | 0.00 | 1 | 0 | 0 | 1 | 5.06 | 4.05 | 4.40 | 5.10 |
| rice_1 | 2 | 1500 | 1500 | **4720** | **2250** | 1.00 | 0.96 | 0.96 | 0.00 | 1.00 | 0 | 0 | 1 | 1 | 3.01 | 2.64 | 3.10 | 3.32 |
| rice_2 | 2 | 1500 | 1500 | **4720** | **2250** | 1.00 | 1.00 | 0.96 | 0.96 | 0.00 | 1 | 0 | 0 | 1 | 3.01 | 2.64 | 3.10 | 3.32 |
| cassava | 6 | 3350 | **10000** | **40000** | **15000** | 0.84 | 1.00 | 0.98 | 0.98 | 0.98 | 1 | 0 | 1 | 1 | 1.36 | 1.26 | 1.40 | 1.41 |
| yam | 6 | 9400 | 9400 | **40000** | **14100** | 0.86 | 0.94 | 0.94 | 0.00 | 1.00 | 1 | 1 | 1 | 1 | 1.54 | 1.73 | 1.90 | 1.56 |
| cocoyam | 6 | 6490 | **10000** | **40000** | **15000** | 0.86 | 0.94 | 0.94 | 0.00 | 1.00 | 1 | 0 | 1 | 1 | 1.54 | 1.73 | 1.90 | 1.56 |
| plantain | 6 | 10900 | 10900 | **40000** | **16350** | 0.69 | 0.94 | 0.94 | 0.00 | 1.00 | 1 | 1 | 1 | 1 | 1.54 | 1.73 | 1.90 | 1.56 |
| sweetpotatoes_1 | 6 | 1870 | **10000** | **40000** | **15000** | 0.75 | 0.00 | 1.00 | 0.94 | 0.94 | 1 | 1 | 0 | 0 | 1.54 | 1.73 | 1.90 | 1.56 |
| sweetpotatoes_2 | 6 | 1870 | **10000** | **40000** | **15000** | 0.75 | 0.94 | 0.00 | 1.00 | 0.94 | 0 | 1 | 1 | 0 | 1.54 | 1.73 | 1.90 | 1.56 |
| cowpea_1 | 3 | 1230 | 1230 | **2272** | **1845** | 1.00 | 0.82 | 0.82 | 0.00 | 1.00 | 0 | 0 | 1 | 1 | 5.52 | 4.42 | 4.80 | 5.57 |
| cowpea_2 | 3 | 1230 | 1230 | **2272** | **1845** | 1.00 | 1.00 | 0.82 | 0.82 | 0.00 | 1 | 0 | 0 | 1 | 5.52 | 4.42 | 4.80 | 5.57 |
| pigeonpea_1 | 3 | 1200 | 1200 | **2218** | **1800** | 1.00 | 0.82 | 0.82 | 0.00 | 1.00 | 0 | 0 | 1 | 1 | 1.96 | 1.56 | 1.70 | 1.97 |
| pigeonpea_2 | 3 | 1200 | 1200 | **2218** | **1800** | 1.00 | 1.00 | 0.82 | 0.82 | 0.00 | 1 | 0 | 0 | 1 | 1.96 | 1.56 | 1.70 | 1.97 |
| bambaragroundnut_1 | 4 | 700 | 700 | **1432** | **1050** | 1.00 | 0.95 | 0.95 | 0.00 | 1.00 | 0 | 0 | 1 | 1 | 6.40 | 5.61 | 6.60 | 7.06 |
| bambaragroundnut_2 | 4 | 700 | 700 | **1432** | **1050** | 1.00 | 1.00 | 0.95 | 0.95 | 0.00 | 1 | 0 | 0 | 1 | 6.40 | 5.61 | 6.60 | 7.06 |
| neri | 4 | 140 | 140 | 140 | 140 | 1.00 | 1.00 | 0.90 | 0.90 | 0.90 | 1 | 0 | 0 | 1 | 4.17 | 3.55 | 3.90 | 4.41 |
| bongu | 4 | 140 | 140 | 140 | 140 | 1.00 | 1.00 | 0.90 | 0.90 | 0.90 | 1 | 1 | 1 | 1 | 7.60 | 6.46 | 7.10 | 8.02 |
| cashewnut | 4 | 560 | 560 | 560 | 560 | 1.00 | 1.00 | 0.90 | 0.90 | 0.90 | 1 | 1 | 1 | 1 | 7.60 | 6.46 | 7.10 | 8.02 |
| soybean_1 | 5 | 810 | 810 | **4480** | **1215** | 1.00 | 0.82 | 0.82 | 0.00 | 1.00 | 0 | 0 | 1 | 1 | 2.88 | 2.30 | 2.50 | 2.90 |
| soybean_2 | 5 | 810 | 810 | **4480** | **1215** | 1.00 | 1.00 | 0.82 | 0.82 | 0.00 | 1 | 0 | 0 | 1 | 2.88 | 2.30 | 2.50 | 2.90 |
| ayoyoleaves_1 | 9 | 9000 | 9000 | 9000 | **13500** | 0.94 | 0.00 | 0.00 | 1.00 | 0.95 | 0 | 0 | 1 | 0 | 4.95 | 3.96 | 4.30 | 4.30 |
| ayoyoleaves_2 | 9 | 9000 | 9000 | 9000 | **13500** | 0.94 | 0.95 | 0.00 | 0.00 | 1.00 | 0 | 0 | 0 | 1 | 4.95 | 3.96 | 4.30 | 4.30 |
| ayoyoleaves_irrigated1 | 9 | 9000 | 9000 | 9000 | **13500** | 0.94 | 1.00 | 0.95 | 0.00 | 0.00 | 1 | 0 | 0 | 0 | 4.95 | 3.96 | 4.30 | 4.30 |
| ayoyoleaves_irrigated2 | 9 | 9000 | 9000 | 9000 | **13500** | 0.94 | 0.00 | 1.00 | 0.95 | 0.00 | 0 | 1 | 0 | 0 | 4.95 | 3.96 | 4.30 | 4.30 |
| braleaves_1 | 9 | 9000 | 9000 | 9000 | **13500** | 0.94 | 0.00 | 0.00 | 1.00 | 0.95 | 0 | 0 | 1 | 0 | 1.04 | 0.83 | 0.90 | 0.90 |
| braleaves_2 | 9 | 9000 | 9000 | 9000 | **13500** | 0.94 | 0.95 | 0.00 | 0.00 | 1.00 | 0 | 0 | 0 | 1 | 1.04 | 0.83 | 0.90 | 0.90 |
| braleaves_irrigated1 | 9 | 9000 | 9000 | 9000 | **13500** | 0.94 | 1.00 | 0.95 | 0.00 | 0.00 | 1 | 0 | 0 | 0 | 1.04 | 0.83 | 0.90 | 0.90 |
| braleaves_irrigated2 | 9 | 9000 | 9000 | 9000 | **13500** | 0.94 | 0.00 | 1.00 | 0.95 | 0.00 | 0 | 1 | 0 | 0 | 1.04 | 0.83 | 0.90 | 0.90 |
| amaranthusleaves_1 | 9 | 9000 | 9000 | 9000 | **13500** | 0.94 | 0.00 | 0.00 | 1.00 | 0.95 | 0 | 0 | 1 | 0 | 2.42 | 1.93 | 2.10 | 2.10 |
| amaranthusleaves_2 | 9 | 9000 | 9000 | 9000 | **13500** | 0.94 | 0.95 | 0.00 | 0.00 | 1.00 | 0 | 0 | 0 | 1 | 2.42 | 1.93 | 2.10 | 2.10 |
| amaranthusleaves_irrigated1 | 9 | 9000 | 9000 | 9000 | **13500** | 0.94 | 1.00 | 0.95 | 0.00 | 0.00 | 1 | 0 | 0 | 0 | 2.42 | 1.93 | 2.10 | 2.10 |
| amaranthusleaves_irrigated2 | 9 | 9000 | 9000 | 9000 | **13500** | 0.94 | 0.00 | 1.00 | 0.95 | 0.00 | 0 | 1 | 0 | 0 | 2.42 | 1.93 | 2.10 | 2.10 |
| Baobableaves^e^ | 9 | - | - | - | - | 0.94 | - | - | - | - | - | - | - | - | 1.04 | 0.83 | 0.90 | 0.90 |
| okro_1 | 11 | 23160 | 23160 | 23160 | **34740** | 0.86 | 0.00 | 0.00 | 1.00 | 0.95 | 0 | 0 | 1 | 0 | 1.73 | 1.38 | 1.50 | 1.50 |
| okro_2 | 11 | 23160 | 23160 | 23160 | **34740** | 0.86 | 0.95 | 0.00 | 0.00 | 1.00 | 0 | 0 | 0 | 1 | 1.73 | 1.38 | 1.50 | 1.50 |
| okro_irrigated1 | 11 | 23160 | 23160 | 23160 | **34740** | 0.86 | 1.00 | 0.95 | 0.00 | 0.00 | 1 | 0 | 0 | 0 | 1.73 | 1.38 | 1.50 | 1.50 |
| okro_irrigated2 | 11 | 23160 | 23160 | 23160 | **34740** | 0.86 | 0.00 | 1.00 | 0.95 | 0.00 | 0 | 1 | 0 | 0 | 1.73 | 1.38 | 1.50 | 1.50 |
| tomatoes_1 | 11 | 8300 | 8300 | 8300 | **12450** | 0.91 | 0.00 | 0.00 | 1.00 | 0.00 | 0 | 0 | 1 | 0 | 2.67 | 3.30 | 3.30 | 3.30 |
| tomatoes_2 | 11 | 8300 | 8300 | 8300 | **12450** | 0.91 | 0.00 | 0.00 | 0.00 | 1.00 | 0 | 0 | 0 | 1 | 2.67 | 3.30 | 3.30 | 3.30 |
| tomatoes_irrigated1 | 11 | 8300 | 8300 | 8300 | **12450** | 0.91 | 1.00 | 0.00 | 0.00 | 0.00 | 1 | 0 | 0 | 0 | 2.67 | 3.30 | 3.30 | 3.30 |
| tomatoes_irrigated2 | 11 | 8300 | 8300 | 8300 | **12450** | 0.91 | 0.00 | 1.00 | 0.00 | 0.00 | 0 | 1 | 0 | 0 | 2.67 | 3.30 | 3.30 | 3.30 |
| onion_leaves_1 | 11 | 9220 | 9220 | 9220 | **13830** | 0.94 | 0.00 | 0.00 | 1.00 | 0.95 | 0 | 0 | 1 | 0 | 4.78 | 5.90 | 5.90 | 4.84 |
| onion_leaves_2 | 11 | 9220 | 9220 | 9220 | **13830** | 0.94 | 0.95 | 0.00 | 0.00 | 1.00 | 0 | 0 | 0 | 1 | 4.78 | 5.90 | 5.90 | 4.84 |
| onion_leaves_irrigated1 | 11 | 9220 | 9220 | 9220 | **13830** | 0.94 | 1.00 | 0.95 | 0.00 | 0.00 | 1 | 0 | 0 | 0 | 4.78 | 5.90 | 5.90 | 4.84 |
| onion_leaves_irrigated2 | 11 | 9220 | 9220 | 9220 | **13830** | 0.94 | 0.00 | 1.00 | 0.95 | 0.00 | 0 | 1 | 0 | 0 | 4.78 | 5.90 | 5.90 | 4.84 |
| cucumber_1 | 12 | 13820 | 13820 | 13820 | **20730** | 0.71 | 0.00 | 0.00 | 1.00 | 0.00 | 0 | 0 | 1 | 0 | 2.67 | 3.30 | 3.30 | 2.71 |
| cucumber_2 | 12 | 13820 | 13820 | 13820 | **20730** | 0.71 | 0.00 | 0.00 | 0.00 | 1.00 | 0 | 0 | 0 | 1 | 2.67 | 3.30 | 3.30 | 2.71 |
| cucumber_irrigated1 | 12 | 13820 | 13820 | 13820 | **20730** | 0.71 | 1.00 | 0.00 | 0.00 | 0.00 | 1 | 0 | 0 | 0 | 2.67 | 3.30 | 3.30 | 2.71 |
| cucumber_irrigated2 | 12 | 13820 | 13820 | 13820 | **20730** | 0.71 | 0.00 | 1.00 | 0.00 | 0.00 | 0 | 1 | 0 | 0 | 2.67 | 3.30 | 3.30 | 2.71 |
| pepper_1 | 12 | 9300 | 9300 | 9300 | **13950** | 0.82 | 0.00 | 0.00 | 1.00 | 0.00 | 0 | 0 | 1 | 0 | 3.30 | 3.30 | 3.30 | 2.71 |
| pepper_2 | 12 | 9300 | 9300 | 9300 | **13950** | 0.82 | 0.00 | 0.00 | 0.00 | 1.00 | 0 | 0 | 0 | 1 | 3.30 | 3.30 | 3.30 | 2.71 |
| pepper_irrigated1 | 12 | 9300 | 9300 | 9300 | **13950** | 0.82 | 1.00 | 0.00 | 0.00 | 0.00 | 1 | 0 | 0 | 0 | 3.30 | 3.30 | 3.30 | 2.71 |
| pepper_irrigated2 | 12 | 9300 | 9300 | 9300 | **13950** | 0.82 | 0.00 | 1.00 | 0.00 | 0.00 | 0 | 1 | 0 | 0 | 3.30 | 3.30 | 3.30 | 2.71 |
| onion_1 | 12 | 18830 | 18830 | 18830 | **28245** | 0.90 | 0.00 | 0.00 | 1.00 | 0.95 | 0 | 0 | 1 | 0 | 4.37 | 5.40 | 5.40 | 4.43 |
| onion_2 | 12 | 18830 | 18830 | 18830 | **28245** | 0.90 | 0.95 | 0.00 | 0.00 | 1.00 | 0 | 0 | 0 | 1 | 4.37 | 5.40 | 5.40 | 4.43 |
| onion_irrigated1 | 12 | 18830 | 18830 | 18830 | **28245** | 0.90 | 1.00 | 0.95 | 0.00 | 0.00 | 1 | 0 | 0 | 0 | 4.37 | 5.40 | 5.40 | 4.43 |
| onion_irrigated2 | 12 | 18830 | 18830 | 18830 | **28245** | 0.90 | 0.00 | 1.00 | 0.95 | 0.00 | 0 | 1 | 0 | 0 | 4.37 | 5.40 | 5.40 | 4.43 |
| palmnut_pulp^f^ | 12 | - | - | - | - | 1.00 | - | - | - | - | - | - | - | - | 1.71 | 1.46 | 1.60 | 1.60 |
| eggplant_1 | 12 | 8880 | 8880 | 8880 | **13320** | 0.81 | 0.00 | 0.00 | 1.00 | 0.00 | 0 | 0 | 1 | 0 | 3.30 | 3.30 | 3.30 | 2.71 |
| eggplant_2 | 12 | 8880 | 8880 | 8880 | **13320** | 0.81 | 0.00 | 0.00 | 0.00 | 1.00 | 0 | 0 | 0 | 1 | 3.30 | 3.30 | 3.30 | 2.71 |
| eggplant_irrigated1 | 12 | 8880 | 8880 | 8880 | **13320** | 0.81 | 1.00 | 0.00 | 0.00 | 0.00 | 1 | 0 | 0 | 0 | 3.30 | 3.30 | 3.30 | 2.71 |
| eggplant_irrigated2 | 12 | 8880 | 8880 | 8880 | **13320** | 0.81 | 0.00 | 1.00 | 0.00 | 0.00 | 0 | 1 | 0 | 0 | 3.30 | 3.30 | 3.30 | 2.71 |
| melon_yellow | 13 | 15400 | 15400 | 15400 | 15400 | 0.51 | 1.00 | 0.00 | 0.00 | 0.00 | 1 | 0 | 0 | 1 | 1.30 | 1.60 | 1.60 | 1.60 |
| watermelon_1 | 13 | 26100 | 26100 | 26100 | 26100 | 0.51 | 0.00 | 0.00 | 0.00 | 1.00 | 0 | 0 | 1 | 1 | 0.46 | 0.50 | 0.50 | 0.49 |
| watermelon_2 | 13 | 26100 | 26100 | 26100 | 26100 | 0.51 | 1.00 | 0.00 | 0.00 | 0.00 | 1 | 0 | 0 | 1 | 0.46 | 0.50 | 0.50 | 0.49 |
| watermelon_irrigated1 | 13 | 26100 | 26100 | 26100 | 26100 | 0.51 | 0.00 | 1.00 | 0.00 | 0.00 | 1 | 1 | 0 | 0 | 0.46 | 0.50 | 0.50 | 0.49 |
| watermelon_irrigated2 | 13 | 26100 | 26100 | 26100 | 26100 | 0.51 | 0.00 | 0.00 | 1.00 | 0.00 | 0 | 1 | 1 | 0 | 0.46 | 0.50 | 0.50 | 0.49 |
| sheafruit_pulp | 13 | 770 | 770 | 770 | 770 | 0.74 | 0.95 | 0.00 | 0.00 | 1.00 | 1 | 1 | 1 | 1 | 0.10 | 0.10 | 0.10 | 0.10 |
| orange_1 | 13 | 19900 | 19900 | 19900 | 19900 | 0.74 | 0.00 | 0.00 | 0.00 | 1.00 | 1 | 1 | 1 | 1 | 1.20 | 1.30 | 1.30 | 1.26 |
| orange_2 | 13 | 19900 | 19900 | 19900 | 19900 | 0.74 | 1.00 | 0.00 | 0.00 | 0.00 | 1 | 1 | 1 | 1 | 1.20 | 1.30 | 1.30 | 1.26 |
| mango_1 | 13 | 7250 | 7250 | 7250 | 7250 | 0.71 | 0.00 | 0.00 | 1.00 | 0.00 | 1 | 1 | 1 | 1 | 1.84 | 1.84 | 1.60 | 1.60 |
| mango_2 | 13 | 7250 | 7250 | 7250 | 7250 | 0.71 | 0.00 | 0.00 | 0.00 | 1.00 | 1 | 1 | 1 | 1 | 1.84 | 1.84 | 1.60 | 1.60 |
| papaya_1 | 13 | 19395 | 19395 | 19395 | 19395 | 0.62 | 0.00 | 0.00 | 1.00 | 0.00 | 1 | 1 | 1 | 1 | 1.84 | 1.84 | 1.60 | 1.60 |
| papaya_2 | 13 | 19395 | 19395 | 19395 | 19395 | 0.62 | 0.00 | 0.00 | 0.00 | 1.00 | 1 | 1 | 1 | 1 | 1.84 | 1.84 | 1.60 | 1.60 |
| tomato_paste_concentrated | 10 | - | - | - | - | 1.00 | - | - | - | - | - | - | - | - | 18.3 | 18.3 | 18.3 | 18.3 |
| vegetable_oil_fortified | 1 | - | - | - | - | 1.00 | - | - | - | - | - | - | - | - | 11.8 | 11.8 | 11.8 | 11.8 |
| small_fish_with_bones | 7 | - | - | - | - | 0.84 | - | - | - | - | - | - | - | - | 12.0 | 12.0 | 12.0 | 12.0 |
| eggs | 8 | - | - | - | - | 0.88 | - | - | - | - | - | - | - | - | 9.50 | 9.50 | 9.50 | 9.50 |
| Fluid powdered_milk_fortified | 14 | - | - | - | - | 1.00 | - | - | - | - | - | - | - | - | 8.70 | 8.70 | 8.70 | 8.70 |

*^a^Average yields with adjusted yields for starchy roots (cassava, cocoyam, sweet potatoes* (van Vugt & Franke, 2018) *that are assumed more realistic than reported averages for Karaga and for Ghana.
^b^Best yields are either modelled best water limited yields or yields based on best attainable yields found, both corrected for the fact that most average farm yields often begin to plateau when they reach 80% of best yields* (Cassman et al., 2003; Lobell et al., 2009) *^c^50% increased yields based on average yields.
^d^Availability for storage scenario for all leafy vegetables (ayoyo, bra, amaranth and onion leaves): assumed 0.95 availability outside of season that is harvested (drying of leaves).  ^e^Baobab leaves are not actively grown, ‘picked from the wild’ but can be bought from the market.
^f^Palmnuts are produced in the south of Ghana (very rare in the North) but can be bought on the market.*

## Supplementary material 6

**Mathematical description of models**

Indices

*s* = seasons (1-4)

*f* = food groups (1-14)

*c* = crops (names used)

Data (coefficients)

*Area_needed_croppingcycle_s,c_* = Land occupation per season, per crop: [1=yes, 0=no]

*Food_availability_season_s,c_* = Yield correction for storage losses per season, per crop, fraction [-]

*Food_prices_s,c_* = Price per season, per crop: [GH₵/kg]

*Foodgroup_requirement_f_* = Food requirements per food group: [kg/season]

*No_food_in_season_s,f,c_* = Food availability per season, per food group, per crop [0= crop not available, >0=crop available]

*On_farm_food_availability_f,s_* = Crop available per food group, per season [1=yes, 0=no]

*Valid_foodgroup_crop_combi_f c_* = Membership of a crop in a food group [1=yes, 0=no]

*Waste_factor_c_* = Yield correction for wastes per crop, fraction [-]

*Yield_c_* = Yield per crop [kg/ha]

In the equation as given in the model descriptions below the names of the binary data (yes/no) are not included but indicated below the equation for matters of readability.

Variables

*Total_area* = Total farm size [ha]

*Total_area_season_s_ =* Land area occupied per season [ha]

*Revenue =* Total farm income from own produce minus costs of foods purchased [GH₵]

*Xarea_s,f,c_* = Land area allocated to production per season, per food group, per crop [ha]

*Crop_produced_s,f,c_* = Production per season, per food group, per crop [kg]

*Crop_consumed_from_farming_s,f,c_* = Consumption from own production per season, per food group, per crop [kg]

*Crop_purchased_s,f,c_* = Food purchased per season, per food group, per crop [kg]

*Cost_crop_purchased_s_* = Costs of crops purchased per season [GH₵]

*Non_crop_purchased_s,f,c_*  = Non-crop foods purchased per season, per food group, per ‘crop’ [kg]

*Cost_non_crop_purchased_s_ = C*osts of non-crop foods purchased per season [GH₵]

*Value_total_crop_sold_f,c_* = Monetary value of own produce sold per food group, per crop: [GH₵]

**Model1: Minimize farm size, priority for food needs covered by own production**

Objective

Minimise crop land:

Minimise (*Total_Area*) [ha] (1)

Subject to

Sum of the area allocated per season is smaller or equal to total the total land area:

$\sum_{s} {Total\_area\_season}_{s}\leq Total\_area$ [ha] (2)

The total area allocated per season cannot exceed the sum of the area allocated to each food group-crop combination, for all food groups produced on-farm:

$\sum_{f, c} {Xarea}_{s,f,c}\leq{Total\_area\_season}_{s}$ [ha] (3)

∀ *f* produced on-farm *and* ∀ valid *f*,*c*-combinations

The area occupied by a crop in a food group is equal to area allocated to it in season 1, if the crop is available in season 1, for all food groups produced on-farm and for all valid food group-crop combinations :

${Xarea}_{s,f,c}={Xarea}_{s=1,f,c}$ [ha] (4)
∀ *f* produced on-farm *and* ∀ valid *f*,*c*-combinations *and* ∀ *c* using land *and* ∀ *c* available in season 1

This equation is similar for seasons 2,3, and 4.

The total amount of a crop produced per food group is at least equal to the amount of the crop consumed on-farm accounting for waste and storage losses, for all food groups produced on-farm, for all valid food group-crop combinations and for all crops available in the season:

$\sum_{s} {Crop\_produced}_{s,f,c}\geq\sum_{s} {Crop\_consumed\_from\_farming}_{s,f,c}*\left( \frac{1}{{Waste\_factor}_{c}} \right)*(\frac{1}{{Food\_availability\_season}_{s,c}})$ [kg] (5)
∀ *f* produced on-farm *and* ∀ valid *f*,*c*-combinations *and* ∀ *c* available in season

The amount of a crop produced per season equals the yield of the crop per ha times the area allocated per season, for all food groups produced on-farm and for all valid food group-crop combinations and for all crops available in season:

${Crop\_produced}_{s,f,c}={Yield}_{c}{*Xarea}_{s,f,c}$ [kg] (6)

∀ *f* produced on-farm *and* ∀ valid *f*,*c*-combinations *and* ∀ *c* available in season

The amount of crops consumed from on-farm production (for crops available in season) plus amount of crops purchased per food group per season (for crops not available in season) cover the food group requirements per season:

$\sum_{c} {Crop\_consumed\_from\_farming}_{s,f,c} ( c available in season)+\sum_{c} {Crop\_purchased}_{s,f,c} ( c not available in season and cheapest crop from f)\geq{Foodgroup\_requirement}_{f}$ [kg] (7)
∀ *f* produced on-farm *and* ∀ valid *f*,*c*-combinations

and the total food group requirements in a year:

$\sum_{s,c} {Crop\_consumed\_from\_farming}_{s,f,c} ( c available in season)+\sum_{s,c} {Crop\_purchased}_{s,f,c} ( c not available in season\mathrm{and} cheapest crop from f)\geq\sum_{s} {Foodgroup\_requirement}_{f}$ [kg] (8)
∀ *f* produced on-farm *and* ∀ valid *f*,*c*-combinations

Total amount of a crop purchased is zero, for all food groups with crop(s) are available in a season:

${Crop\_purchased}_{s,f,c}=0$ [kg] (9)

∀ *f* where *c* are available in a *s*

The monetary value of a crop sold equals the sum over the seasons of the amount produced times the seasonal price of the crop minus the sum over the seasons of consumption from on-farm production accounting for waste and storage losses times the seasonal price of the crop, for all food groups produced on-farm and for all valid food group-crop combinations and for all crops available in season:

$Value\_total\_{crop\_sold}_{f,c}=(\sum_{s} {Crop\_produced}_{s,f,c}*{Food\_prices}_{c,s}$) - $(\sum_{s} {Crop\_consumed\_from\_farming}_{s,f,c}*\left( \frac{1}{{Waste\_factor}_{c}} \right)*(\frac{1}{{Food\_availability\_season}_{s,c}}){*Food\_prices}_{c,s})$ [GH₵] (10)
∀ valid *f*,*c*-combinations  *and* ∀ *c* available in season

The costs of crops purchased per season equals the sum of crops purchased per food group per season times the seasonal price of the crop, for all food groups produced on the farm and for all valid food group-crop combinations and for cheapest crop available in a food group:

${Cost\_crop\_purchased}_{s}=\sum_{f,c} {Crop\_purchased}_{s,f,c}* {Food\_prices}_{c,s}$ [GH₵] (11)
∀ *f* produced on-farm *and* ∀ valid *f*,*c*-combinations *and* $cheapest crop from f$

The amount of non-crop foods purchased per season per food group equals the food group requirements, for all food groups never produced on-farm and for all valid food group-‘crop’ combinations and for cheapest ‘crop’ available in a food group:

${Crop\_purchased}_{s,f,c}$ *=* ${Foodgroup\_requirement}_{f}$ [GH₵] (12)
∀ *f* not produced on-farm *and* ∀ valid *f*,*c*-combinations *and* $cheapest 'crop' from f$

The costs of non-crop foods purchased per season equal the sum of foods purchased per food group times the seasonal price of the ‘crop’, for all food groups never produced on-farm and for all valid food group-‘crop’ combinations and for cheapest ‘crop’ available in a food group:

${Costs\_non\_crop\_purchased\_}_{s}=\sum_{f,c} {Crop\_purchased}_{s,f,c}* {Food\_prices}_{c,s}$ [GH₵] (13)
∀ *f* not produced on-farm *and* ∀ valid *f*,*c*-combinations *and* $cheapest 'crop' from f$

The total costs of foods purchased equals the sum of costs of crops purchased and cost of non-crop foods purchased, for all valid food group and crop combinations:

$Total\_costs\_food\_purchased= \sum_{s} {(Cost\_crop\_purchased}_{s}+ {Costs\_non\_crop\_purchased}_{s})$

[GH₵] (14)

Total value of crops sold per food group equals the total costs of food purchased, for all valid food group-crop combinations:

$\sum_{f,c} {Value\_total\_crop\_sold}_{f,c}= Total\_costs\_food\_purchased$ [GH₵] (15)

∀ valid *f*,*c*-combinations

The revenue equals the sum of total value of crops sold per food group minus costs of foods purchased:

*Revenue* = $\sum_{f,c} {Value\_total\_crop\_sold}_{f,c}-Total\_costs\_food\_purchased$ [GH₵] (16)

∀ valid *f*,*c* combinations

**Model 2: Maximize revenue, priority for food needs covered by own production**

Objective

Maximise revenue:
Maximise (*Revenue =* $\sum_{f,c} {Value\_total\_crop\_sold}_{f,c}-Total\_costs\_food\_purchased$) [GH₵] (17)

∀ valid *f*,*c* combinations

Subject to
Equations are similar to Model 1, except that this model is **not subject to** equation (15) - the value of crops sold equals the purchases - and in addition **is subject to**:

$$\sum_{f,c} {Xarea}_{s,f,c} \leq$$

 *Total_Area* (outcome from model1) [ha] (18)

**Model 3: Maximize revenue, no priority for food needs covered by own production**

Objective

Maximise revenue similar to equation (17)

Subject to

Equations are similar to Model 2, except this model is **not subject** to equation (9) - crop are not purchased if they can be produced on farm.

**Model 4: Minimize farm size, no priority for food needs covered by own production**

Objective

Minimise crop land:

Minimise (*Total_Area*)

Subject to

Equations are similar to Model 1, except this model is **not subject** to equation (9) - crop are not purchased if they can be produced on farm.

## Supplementary material 7

**Maximum revenue^ for an average household in Northern Ghana with priority to cover food needs by own production (scenario A),**

**for 100% and 80% of the prices assumed**

|  | **Farm size cultivated (ha)** | | **Maximum revenue**  **(100% prices)** | | | **Maximum revenue**  **(80% prices)** | | |
| --- | --- | --- | --- | --- | --- | --- | --- | --- |
|  |  | | ***absolute*** | ***relative*** | | ***absolute*** | ***relative*** | |
|  | *S1, S3, S4* | *S2* | *GH₵/year* | *GH₵/year* | *GH₵/year/ha* | *GH₵/year* | *GH₵/year* | *GH₵/year/ha* |
| 1. Average yields | 1.43 | 0.26 | 116600 | 116600  (100%) | 81538  (100%) | 93300 | 93282  (100%) | 100% |
|  |  |  |  |  |  |  |  | |
| 2. Expand availability^a^ |  |  |  |  |  |  |  |  |
| a. Storage^b^ | 1.41 | 0.33 | 102900 | 88% | 90% | 82300 | 88% | 89% |
| b. Irrigation | 1.43 | 1.43 | 264200 | 227% | 227% | 211400 | 227% | 227% |
| 3. Improved crop yields |  |  |  |  |  |  |  |  |
| a. Best yields^c^ |  |  |  |  |  |  |  |  |
| grains | 1.02 | 0.38 | 62700 | 54% | 75% | 50200 | 54% | 75% |
| starchy roots | 1.43 | 0.26 | 129000 | 111% | 111% | 103200 | 111% | 111% |
| legumes | 1.00 | 0.22 | 75500 | 65% | 93% | 60400 | 65% | 93% |
| b. 50% higher yields^d^ |  |  |  |  |  |  |  |  |
| grains | 1.24 | 0.31 | 91100 | 78% | 90% | 72908 | 78% | 90% |
| starchy roots | 1.43 | 0.26 | 118700 | 102% | 102% | 94934 | 102% | 102% |
| legumes | 1.15 | 0.28 | 84000 | 72% | 90% | 67253 | 72% | 90% |
| vegetables | 1.42 | 0.31 | 164400 | 141% | 142% | 131498 | 141% | 142% |

*^Maximal revenue is the monetary value of own production minus the costs of foods purchased (5300 GH₵/year for non-crop foods plus additional 200 GH₵ for vegetables and fruits in Season 2 (5500 GH₵/year), except in case of expanding availability (for storage: 50 GH₵ extra (5350 GH₵/year), for irrigation: none (5300 GH₵/year))
S1=season from November to January (dry period), S2=season from February to April (dry period), S3=season from May to July (rainy period), S4-season from August to October (rainy period)
- =no differences in crops harvested compared with ‘average yields’ scenario*^a^expand availability of vegetables and fruits as we found they cannot be harvested throughout the year
^b^storage includes local feasible options such as drying of vegetable leaves
^c^the largest yields attained in field experiments in a specific area
^d^yields 50% above the average yields
